# The Effectiveness of a Primary School Based Badminton Intervention on Children’s Fundamental Movement Skills

**DOI:** 10.3390/sports8020011

**Published:** 2020-01-21

**Authors:** Michael J. Duncan, Mark Noon, Chelsey Lawson, Josh Hurst, Emma L. J. Eyre

**Affiliations:** Centre for Sport, Exercise and Life Sciences, Coventry University, Coventry CV1 5FB, UK; aa5349@coventry.ac.uk (M.N.); ac2444@coventry.ac.uk (C.L.); ac2050@coventry.ac.uk (J.H.); ab2223@coventry.ac.uk (E.L.J.E.)

**Keywords:** physical education, motor skill, motor competence, test of gross motor development, object-control

## Abstract

This study examined the effects of the Badminton World Federation (BWF) Shuttle Time program on fundamental movement skills (FMS) in English children. A total of 124 children; 66 in key stage 1 (ages 6–7 years) and 58 in key stage 2 (10–11 years) undertook the Shuttle Time program, once weekly for six weeks (n = 63) or acted as controls (n = 61). Pre, post and ten-weeks post, both process and product FMS were determined. Children in the intervention group, aged 6–7 years, had higher total process FMS (via test of gross motor development-2) compared to the control group at post and ten-weeks post intervention (both *p* = 0.0001, d = 0.6 and 0.7, respectively). There were no significant differences in process FMS scores for children aged 10–11 years. Ten-meter sprint speed decreased pre to post and was maintained at ten-weeks post for the intervention groups aged 6–7 years (*p* = 0.0001, d = 0.6) and 10–11 years (*p* = 0.001, d = 0.2) compared to control. Standing long jump distance increased pre to post (*p* = 0.0001, d = 0.8) and was maintained at ten-weeks post (*p* = 0.0001, d = 0.5) for the intervention group. Medicine ball throw performance increased pre to post (*p* = 0.0001, d = 0.3) for the intervention group. The BWF Shuttle Time program is beneficial in developing FMS for key stage 1 children (ages 6–7).

## 1. Introduction

Fundamental movement skills (FMS) refer to an aspect of motor competence considered to be the building blocks that lead to specialized movement sequences required for adequate participation in organized and non-organized sports and physical activities [1]. Globally defined as locomotor (e.g., running, jumping), object control (e.g., throwing, catching), and stability (e.g., balancing and twisting) skills [2,3]. There has been increasing research interest on the topic of FMS development in children [4,5] as FMS are conceptualized to play an important role in opportunities to lead an active lifestyle and the maintenance of healthy weight status during childhood [6]. Stodden et al. [6] developed a conceptual model and posited that in middle and late childhood, motor competence—through FMS—directly leads to physical activity which, in turn, influences weight status. This is unlike early childhood where the relationship between motor competence and physical activity is proposed to be reciprocal [6]. Such assertions have been empirically supported for both weight status [7] and physical activity [5].

As a consequence, the development of FMS, either in isolation or as part of the development of physical literacy, has therefore become prominent in school physical education curricula worldwide [8,9,10]. In the context of England, the most recent changes to the physical education curriculum explicitly identify the development of FMS as key outcomes within key stage 1 (ages 5–7 years), and the development of fundamental sports skills as key within key stage 2 (7–11 years). Yet, there is concern that FMS competence in British children is low [11] and there have been calls to trial effective interventions to better develop FMS during the primary school period (ages 5–11 years). There is also an acknowledgement in the literature that FMS differs according to sex [1,4,5] and thus potential differences in FMS between boys and girls need to be considered when examining this issue. It is important to note that age plays a key role in FMS development where FMS are posited to develop from the ages of two to seven years and, as proficiency develops these FMS are combined and refined in a variety of ways to develop fundamental sport skills [12]. This point is reflected in the expectations of the national curriculum for physical education in England but also highlights that the effect of FMS-related interventions may differ for children in the different key stages of the English curriculum. However, while these curricula milestones suggest when FMS and fundamental sports skills should be developed, data suggest that British children may not master their FMS at these curricular milestones [11] and the acquisition of FMS should be considered a continuum where skills can be learned and refined across different stages of physical education in primary schools [12].

A variety of intervention models have been trialed with school children with a view to enhancing children’s FMS (e.g., [5,11,13,14]). These aforementioned interventions have had success but largely focus on practice of FMS skills in isolation and without the context of sport performance. In a curriculum which emphasizes a transition of developing FMS into fundamental sports skills, providing a sport-based context would seem to be pragmatic and aligned to the curriculum. It is also important to differentiate the effects of FMS-based interventions between key stages within the English school curriculum in order to better guide teaching practice. No studies to date have examined if the effects of FMS intervention differ as a result of which key stage of the curriculum it is delivered in.

One established intervention which may be particularly appealing for use in school physical education, the Badminton World Federation (BWF) Shuttle Time initiative [15], provides a program which aims to achieve the objectives of physical education through badminton-related activities for children aged 5–15 years. The intervention claims to be developmentally tailored and is aligned with the demands of school curriculum worldwide [15]. Implicit within the activities included in the program are the development of FMS which, although badminton-related, also apply to a range of sports and physical activities. Within the Shuttle Time program, although both locomotor and object control skills are developed, there is a predominant focus on the development of object control skills [15]. Such a focus is important as the development of object control skills has been considered as more important than locomotor skills for overall motor development [16]. This is because object control skills have greater skill component complexity and perceptual demand than locomotor skills, thus requiring more intensive skill instruction and practice [16]. Developing object control skills has also been identified as an important avenue for increasing overall FMS and physical activity [17], and an explicit focus more on object control skills in physical education may be effective in positively enhancing the different components of the Stodden et al. [6] conceptual model to a greater extent than statutory physical education. Likewise, although other racquet sports such as tennis and table tennis likely will involve the development of object control skills, other than the BWF Shuttle Time program, there is currently no other racquet sport-based intervention purported to enhance FMS in school children and, although anchored in badminton, the activities involved are purported to develop general FMS as well as those used in all racquet sports [15]. However, no study to date has determined if the BWF Shuttle Time intervention is effective in enhancing FMS in children, despite the fact that it is currently employed in over 120 countries [15]. Given the extent to which the BWF Shuttle Time program is used across the world but that, to date, the efficacy of the program remains uninvestigated, a key step to ensure evidence-based practice in schools is to determine if the Shuttle Time program is effective for those children who engage with it.

The current study addresses a gap in the literature by examining the following research question: Is a six-week BWF Shuttle Time intervention effective in enhancing primary outcome variables of process and product FMS in children? The examination of both process and product measures of FMS is important as authors have advocated the more holistic assessment of FMS to include both process (reflecting the quality of movement) and product (reflecting the outcome of the movement) measures of FMS [18]. Given that the BWF Shuttle Time program is advocated for use in children age 5–15 years [15] and the focus on development of FMS into fundamental sports skills in the key stage 1 and 2 physical education curriculum, a secondary aim in the present study was to investigate whether the effect of a six-week BWF Shuttle Time intervention differed when administered in children in key stage 1 (ages 6–7 years) and key stage 2 (10–11 years) of the English school curriculum.

## 2. Materials and Methods

### 2.1. Design

This study employed a repeated measures approach, with a cluster randomized intervention design where four classes from two schools in central England were allocated into two conditions: (1) Shuttle Time intervention (INT); (2) control (CON) groups. The schools involved were comparable in terms of ethnic makeup and were all within the mid-range of socio-economic status for the county in which they were based. The children were drawn from one school for year 2 children (ages 6–7) and another school for year 6 (ages 10–11) children. Each class in each school were subsequently split into intervention (year 2, n = 34, year 6 n = 29) and control groups (year 2, n = 32, year 6 n = 28) (i.e., intervention and control groups were drawn from the same school). Allocation of classes to INT or CON conditions was done by chance (coin toss). In this way we sought to not only evaluate the effect of the Shuttle Time intervention compared to the control group but also to examine if the effects of the intervention differed depending on stage of childhood from early primary school to late primary school. The INT groups undertook a structured Shuttle Time program over a six-week period in place of one (of the two) statutory physical education sessions and lasted 60 min with 50 min being spent as ‘time on task.’ Full guidance on how the Shuttle Time program was administered is presented in the BWF Shuttle Time Teacher Manual [15]. Shuttle Time children therefore engaged in one Shuttle Time and one physical education session per week. The CON group did not perform specific FMS intervention but attended their two statutory physical education classes per week. The physical education activities engaged in by the groups were the same and consisted of cricket, a sport also requiring object control skills. Prior to, immediately following, and 10 weeks post the intervention, participants in both groups were assessed on measures of process and product FMS and weight status. The choice of a 10-week post intervention testing timescale was pragmatic for the school timetable where the intervention took place in one school term and the post intervention testing took place at the end of the next school term.

### 2.2. Participants

One hundred and twenty-four children aged 6–11 years (67 boys, 57 girls; Mean ± SD = 8.5 ± 1.9 years) from two central England primary schools participated in this study following institutional ethics approval, written informed parental consent, and child assent. Participants were drawn from two classes in school year 2 (n = 66, ages 6–7, key stage 1) or from two classes in school year 6 (n = 58, ages 10–11, key stage 2). From school records, ethnic classifications of these participants were: 95% ‘Caucasian’; 2% ‘South Asian’; 3% ‘Black’. The schools were selected using convenience sampling; they were located in areas ranked as 40–60% least deprived within England as a whole, using the Index of Multiple Deprivation [19]. Inclusion criteria comprised being a child in either school year 2 or 6 in the schools taking part and not having a registered special educational need or any form of musculoskeletal issue that would impair movement.

### 2.3. Measures

Process and product measurements of FMS were employed in the present study. Process oriented movement skill assessment are concerned with how the skill is performed [20] whereas product measures are concerned with the outcome of the movement [18]. In this way we sought to provide a holistic evaluation of FMS in line with suggestions made by Logan et al. [18].

### 2.4. Process Fundamental Movement Skills (FMS)

Five tasks (two locomotor, three object control) were employed to assess process FMS assessed using the test of gross motor development-2 (TGMD-2) [21]. In the current study the following skills were assessed: Run, jump, catch, throw, strike. All skills were video recorded (Sony video camera, Sony, UK) and subsequently edited into single film clips of individual skills on a computer using Quintic Biomechanics analysis software v21 (Quintic Consultancy Ltd., Birmingham, UK). Scores from two trials were summed to obtain a raw score for each skill. The scores for all the skills were then summed to create a total motor competence (scored 0–40) score. Scores from the run and jump were summed to create a locomotor competence score (0–16) and the catch, throw, and strike summed to create an object control score (0–24) following recommended guidelines of administration of the TGMD-2 [21]. Two researchers experienced in the assessment of children’s movement skills (having previously assessed movement skills in the context of a previous research study) analyzed the motor competence videos. Congruent with prior research [22], training was considered complete when each observer’s scores for the two trials differed by no more than one unit from the instructor score for each skill (>80% agreement). Intraclass correlation coefficients for inter and intra-rater reliability were 0.925 (95% CI = 0.87–0.95) and 0.987 (95% CI = 0.94–0.98), respectively, demonstrating good reliability [23].

### 2.5. Product FMS

Three product measures of FMS; 10 m flying sprint time, standing long jump (SLJ), and seated medicine ball (1 kg) throw (MBT) were assessed. Although in some instances these measures have been considered as measures of motor fitness, they are also recognized as measures of product FMS in their own right [18] given that motor fitness is key to performance of FMS and the outcome or product of the FMS is reflected by the measures participants undertook. Procedures were congruent with those used previously by Duncan et al. [13] in their evaluation of school-based training interventions. A 10-metre sprint run (s) was timed using Smart Speed gates (Fusion Sport, Coopers Plains, Australia) as per Duncan et al. [13]. Standing long jump (cm) was measured following procedures described by Peterson [24]. The seated MBT was conducted, using a 1 kg medicine ball, as a measure of upper body strength following procedures reported by Davis et al. [25]. The seated MBT has been identified as a reliable and valid measure of upper body strength in children [25]. Children made three attempts to throw the medicine ball in a chest pass motion whilst sat on the floor, with the furthest distance thrown been taken as a measure of upper body strength. All measures of product FMS demonstrated acceptable reliability with intraclass correlation coefficients between 0.86 and 0.94.

### 2.6. Shuttle Time Intervention

The present study employed a six-week version of the BWF Shuttle Time program [15]. No specific optimum duration of the Shuttle Time program is specified by the BWF and a six-week trial period was chosen as, pragmatically, it fitted within a school half term, making it attractive for teachers for potential roll out in multiple schools. This decision was taken to have minimal disturbance on the school curriculum as per other studies [11,16]. No study to date has examined the efficacy of Shuttle Time so delivery and focus of the intervention sessions followed guidance provided by the Badminton World Federation in their Teacher’s Manual [15]. The Shuttle Time program was progressive, based on the exercises and activities specified by the BWF and consisted of a warm-up section (10 min) and a main body section (approximately 40 min). The intervention focused on development of the following: Balance, coordination, underhand throwing, catching, striking, running, jumping, and correct use of a racquet (to grip and swing) [15]. The INT groups also undertook a second weekly physical education (PE) lesson during the intervention period, as part of statutory physical education, which was focused on team games (hockey and basketball). The CON group continued their twice weekly statutory physical education lessons with one weekly session focused on cricket and the other on team games (hockey and basketball). In this way we tried to match the lessons the children undertook so the control group’s statutory PE sessions comprised an object control stimulus in lieu of the Shuttle Time intervention. In this context cricket involves striking, throwing, and locomotor activity so provided as comparable a stimulus to the Shuttle Time activities without it being a racquet sport as well as being a component activity of statutory PE in the national curriculum.

The principal investigators delivered all the intervention sessions with the assistance of a primary school teacher. The other sessions for the Shuttle Time group and the CON group were delivered by the physical education teacher. Any child who missed more than one session in the intervention period was not included in final analysis. This resulted in two exclusions from the final data set for analysis, one from the CON group and one from the Shuttle Time group due to the children moving schools during the intervention period.

Table 1 outlines the content and schedule of the Shuttle Time program. Similar to other research using this approach with children [11,13,14], participants in the intervention groups also received skill-specific feedback on the quality of each movement during intervention sessions. Such feedback took the form of knowledge of performance (i.e., feedback about the movement themselves) and was provided verbally following each activity within each session (i.e., immediate feedback) in line with the guidance provided by BWF in administering the Shuttle Time intervention [15]. Conversely, in the CON group, feedback on performance was not provided.

### 2.7. Statistical Analysis

A series of repeated measures ANOVAs were used to examine any changes in dependent variables; process FMS, the three measures of product FMS, and BMI assessed pre, post and 10 weeks post the intervention period. Group (INT vs. CON), sex, and age group (6–7 years vs. 10–11 years) were used as between-subject variables. In this way we sought to assess any short term (pre-post) and sustained (post-10 weeks post) changes in dependent variables between intervention and control groups, sex groups, and between children in school year 2 and 6. Sex was entered in the model as considerable prior review papers and empirical research has identified that FMS differ between boys and girls [1,4,5,11,17]. At baseline there were no significant differences (*p* > 0.05) for any of the dependent variables between INT and CON groups aged 6–7 or between INT and CON groups aged 10–11 years. Where any differences were found Bonferroni post-hoc analysis was undertaken to determine where differences lay. Partial eta squared (ηp^2^) was used as a measure of effect size with values classified as small (0.01), medium (0.09), and large (0.25), and alpha level was set as *p* = 0.05 to indicate statistical significance. Cohen’s *d* magnitude of effect size is reported for pairwise comparisons and were interpreted as trivial (<0.20), small (0.2), moderate (0.6), large (1.2), and very large (>2.0) [26]. The Statistical Package for Social Sciences (IBM SPSS Statistics for Windows, Version 24.0. Armonk, NY: IBM Corp) was used for all analyses. The coefficient of variation for dependent variables were 15% for total FMS, 9.2% for 10 m sprint speed, 17% for standing long jump, and 18% for the 1 kg seated medicine ball throw at baseline which were comparable with prior studies using similar methods [11,13].

## 3. Results Process FMS

**Figure 1:**
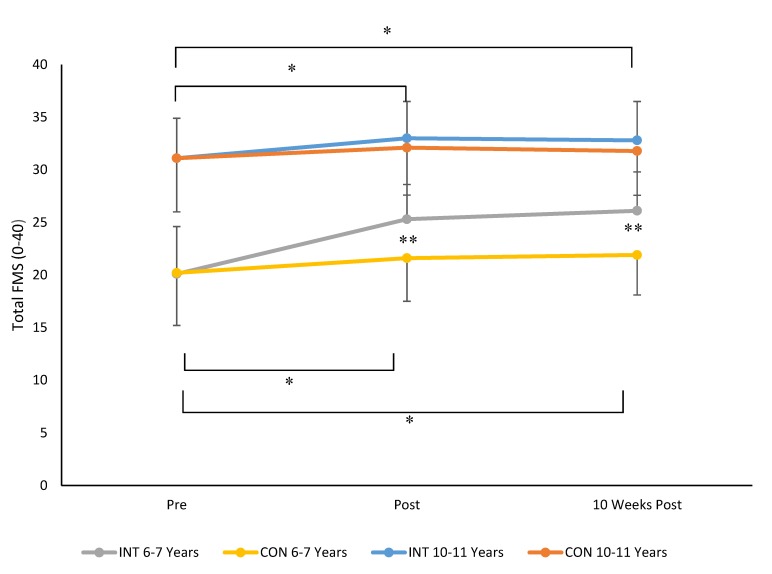
Mean ± SD of total fundamental movement skills (FMS) for children aged 6–7 years and 10–11 years in Shuttle Time intervention (INT) and control (CON) groups (** *p* = 0.0001, * *p* < 0.05).

When data for total FMS scores were considered, results revealed a time × group × age stage interaction (F 2, 230 = 24.6, *p* = 0.001, ηp^2^ = 0.18, see Figure 1). Bonferroni post-hoc analysis indicated that there was no significant difference in total FMS in INT and CON groups aged 6–7 years (*p* = 0.998) or children aged 10–11 years (*p* = 0.978) pre intervention. At post intervention (*p* = 0.0001) and at 10 weeks post intervention (*p* = 0.0001) children aged 6–7 years in the INT group had significantly higher total FMS compared to children aged 6–7 years in the CON group. There were no significant differences in total FMS scores for children aged 10–11 years in INT and CON groups post intervention (*p* = 0.431) and 10 weeks post intervention (*p* = 0.361). For children aged 6–7 years and children aged 10–11 years in INT and CON, total FMS significant increased pre to post intervention (all *p* < 0.05). Total FMS scores at 10 weeks post intervention also remained significantly higher than post for children aged 6–7 years in INT and CON groups and children aged 10–11 years in the INT group (all *p* < 0.05). Total FMS scores only improved significantly from post intervention to 10 weeks post intervention for 6–7-year-old children in the INT group (*p* = 0.03). The magnitude of change in total FMS scores was greatest for children aged 6–7 years in the intervention group with Cohen’s *d* scores indicating a moderate effect for changes in FMS pre to post (*d* = 0.6, moderate) and pre to 10 weeks post (*d* = 0.7, moderate) for 6–7-year-olds who undertook the intervention. In all cases, total FMS scores were higher for children aged 10–11 years compared to those aged 6–7 years, irrespective of group. The magnitude of changes in FMS for 10–11-year-old children was small with Cohen’s *d* values of 0.2 for changes in FMS pre to post and pre to 10 weeks post intervention.

Repeated measures ANOVA also identified significant main effects for sex (F 1, 115 = 14.5, *p* = 0.001). Boys also had significantly higher total FMS scores than girls, irrespective of age stage or group. Mean (±SD) of total FMS was 28.6 (6.2) for boys and 25.9 (6.1) for girls.

### 3.1. Product FMS

#### 3.1.1. Ten-Meter Sprint Speed

**Figure 2:**
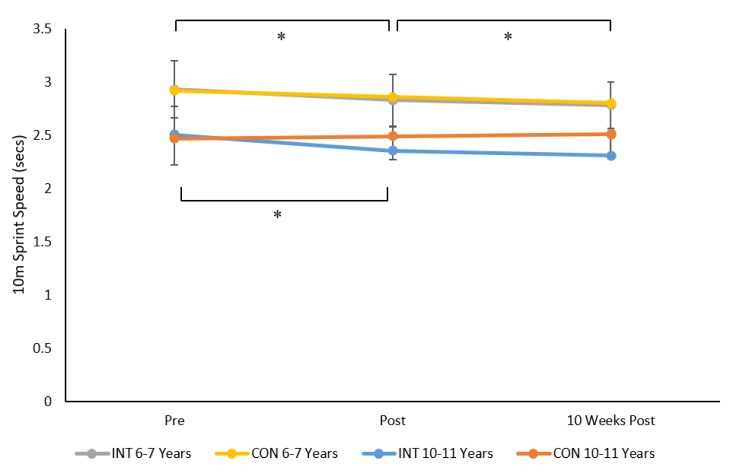
Mean ± SD of 10 m sprint speed across time for the INT and CON groups in children aged 6–7 years and 10–11 years, * *p* =.01

For 10 m sprint speed results indicated a significant time × group × age group interaction (F 2, 230 = 6.5, *p* = 0.002, ηp^2^ = 0.05, see Figure 2). Post-hoc analysis indicated no significant differences between INT and CON group in children aged 6–7 and 10–11 years at pre, post, and 10 weeks post intervention (all *p* > 0.05). However, 10 m sprint time decreased (i.e., performance increased) pre to post for INT groups aged 6–7 years (*p* = 0.0001, *d* = 0.5), 10–11 years (*p* = 0.001, *d* = 0.2), and the CON group aged 6–7 years (*p* = 0.003, *d* = 0.1). This improvement was maintained from pre to 10 weeks post intervention for the aforementioned groups, however sprint speed was only significantly different (*p* = 0.025) from post to 10 weeks post for children aged 6–7 years in the INT group. The Cohen’s *d* value of 0.2 indicated a small effect from post intervention to 10 weeks post for 6–7-year-old children.

There were also significant main effects for sex (F 1, 115 = 14.8, *p* = 0.001, ηp^2^ = 0.12) and age stage (F 1, 115 = 116.3, *p* = 0.001, ηp^2^ = 0.51). Boys and children aged 10–11 years were significantly faster than girls and children aged 6–7 years, respectively. Mean (±SD) of 10 m run speed was 2.57 (0.033) s and 2.7 (0.031) s for boys and girls respectively and 2.85 (0.025) s and 2.4 (0.031) s for children aged 6–7 and 10–11 years, respectively.

#### 3.1.2. Standing Long Jump

**Figure 3:**
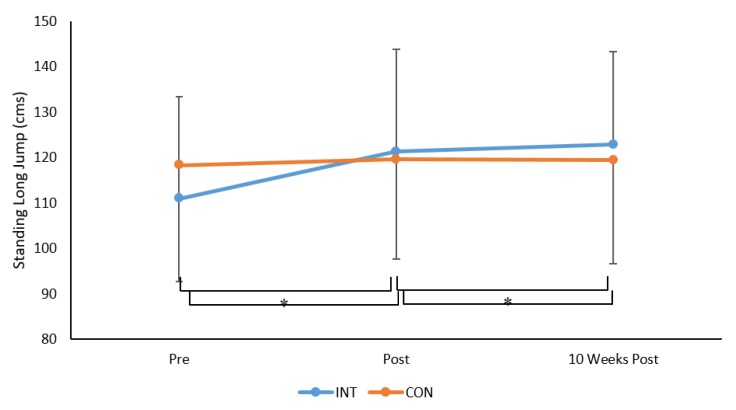
Mean ± SD of standing long jump (cm) across time for the INT and CON groups, * *p* = 0.0001.

For SLJ there was a significant time × group × interaction (F 2, 230 = 19.3, *p* = 0.001, ηp^2^ = 0.15, see Figure 3). Bonferroni post-hoc analysis indicated that SLJ distance increased pre to post for the INT group (*p* = 0.0001, *d* = 0.8, moderate) but not the CON group (*p* = 0.728). Standing long jump scores were also significantly greater at 10 weeks post intervention, compared to post, for the INT group (*p* = 0.0001, *d* = 0.5, small to moderate) but not the CON group (*p* = 0.956), but were not different from post intervention to 10 weeks post intervention for the INT (*p* = 0.306) or CON groups (*p* = 0.737).

Results revealed no main effect due to sex (*p* > 0.05) but there was a significant main effect for age group (F 1, 115 = 47.5, *p* = 0.001, ηp^2^ = 0.29). Irrespective of group, children aged 6–7 years had smaller SLJ scores compared to children aged 10–11 years. Mean (±SD) of SLJ (cm) was 107 (17.9) cm and 130 (21.9) cm for children aged 6–7 years and 10–11 years, respectively.

#### 3.1.3. One-Kilogram Seated Medicine Ball Throw

**Figure 4:**
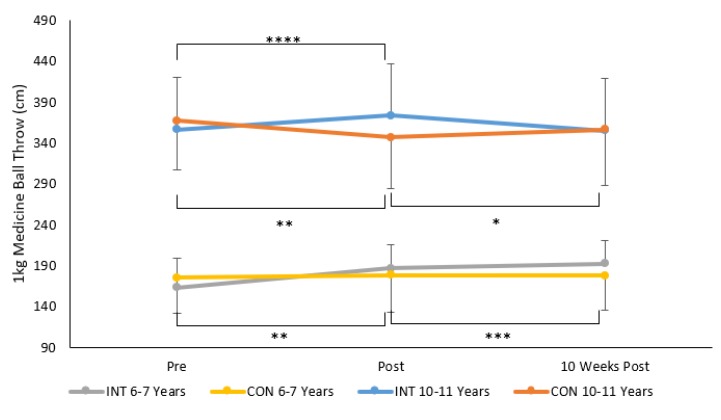
Mean ± SD of 1 kg seated medicine ball throw distance (cm) across time for the INT and CON groups in children aged 6–7 years and 10–11 years, * *p* = 0.027, ** *p* = 0.003, *** *p* = 0.001, **** *p* = 0.0001.

For the 1 kg seated MBT throw, results from repeated measures ANOVA revealed a significant time × group × age group interaction (F 2, 230 = 6.9, *p* = 0.001, ηp^2^ = 0.06, See Figure 4). Bonferroni post-hoc pairwise comparisons indicated that there were no significant differences in MBT distance between the INT and CON groups at pre, post, and 10 weeks post intervention in both the 6–7-year-olds and 10–11-year-olds (all *p* > 0.05). For 6–7-year-olds in the INT group, MBT performance increased pre to post (*p* = 0.001, *d* = 0.3, small) and pre to 10 weeks post (*p* = 0.001, *d* = 0.1, trivial). There were no significant differences in MBT performance pre, post to 10 weeks post for 6–7-year-olds in the CON group. For 10–11-year-olds in the INT group MBT distance significantly increased pre to post (*p* = 0.0001, *d* = 0.1, trivial) and then significantly decreased post intervention to 10 weeks post intervention (*p* = 0.0001, *d* = 0.1, trivial). For 10–11-year-olds in the CON group, MBT distance significantly decreased pre to post (*p* = 0.003, *d* = 0.1, trivial) and then significantly increased post to 10 weeks post (*p* = 0.027, *d* = 0.07, trivial).

Results also indicated a significant main effect for sex (F 1, 115 = 11.6, *p* = 0.001, ηp^2^ = 0.09) and age stage (F 1, 115 = 405.6, *p* = 0.001, ηp^2^ = 0.79) where MBT distance was significantly higher for boys compared to girls and for children aged 10–11 years compared to 6–7 years. Mean (±SD) of seated MBT distance (cm) was 284 (98.3) cm and 253.7 (101.7) cm for boys and girls respectively and 178.8 (38.4) cm and 359.1 (66.5) cm for children aged 6–7 and 10–11 years, respectively.

## 4. Discussion

This study is the first to examine the effects of the BWF Shuttle Time program on process and product FMS in children. We examined short term (pre-post intervention) effects and longer term (post-10 weeks post intervention) effects to provide an indication of the longer-term retention of any change as a result of the program. A key tenant of the Shuttle Time program is the development of competence in FMS that are developed through badminton but are applicable to a range of different sports [15]. The present study supports the assertion that Shuttle Time enhances children’s FMS [15]. The current study represents a novel contribution to the literature as, although Shuttle Time is widely used across the world [12], to date no research had investigated the efficacy of this program. The current study presents this data for the first time alongside data documenting longer term retention of changes following the intervention, an often under-investigated issue.

The results of the current study align with prior work which suggests that school-based movement interventions enhance both process [11,13] and product FMS [13]. To some extent, the results of the present study could have been expected. There are a considerable number of studies which suggest school-based interventions focusing on motor competence enhance children’s FMS when delivered by trained professionals (see [16] for a review), with meta-analytical data suggesting interventions focusing on object control skills are more effective [16]. The results of the present study align with these assertions but suggest the BWF Shuttle Time intervention was more effective for children in key stage 1 rather than key stage 2. The current study differs from prior school-based intervention research [11,13,14] in that the Shuttle Time program, although focused on developing FMS in general, is anchored in a specific sport, rather than being a more generic type program as used by prior work [11,13,14]. However, meta-analytical data [16] have suggested that interventions focusing on object control skills may be of greater benefit to overall FMS development that those focusing more on locomotor skills. As the BWF Shuttle Time program predominantly focuses on object control skills more than locomotor skills, the results presented here would support the assertions from the meta-analysis of Morgan et al. [16].

The activities within the intervention itself comprise directed learning tasks with specific coaching cues and manipulation of task constraints [27], such as use of balloons instead of shuttlecocks or removal of badminton nets during early stages of the intervention, which are considered the most important factor of Newell’s [27] constraints-led approach in developing movement skills [28]. We speculate this approach is the primary mechanism responsible for the improvement in FMS reported in the present study. Although baseline and post intervention process FMS scores were higher for children aged 10–11 years compared to their 6–7-year-old peers, the process FMS scores suggest that the older children were not yet fully competent in the FMS, reinforcing prior concerns [11] that FMS competency in British children is low. The movement patterns for the older children may have been more fully developed compared to the younger children in the current study, in line with assertions made by Gallahue, Ozman, and Goodway [2] in regard to the development of FMS. This, in turn, may have made the intervention less effective in key stage 2 children compered to their key stage 1 peers. Likewise, for older children, an intervention of the frequency (once per week) and duration (six weeks) may not be optimal for enhancement of FMS where movement patterns are less malleable. It is also possible that, for older children, the Shuttle Time program was less attractive or viewed as less physically challenging, compared to the younger age participants, but resulting in less effectiveness of the program for older children.

Although no prior work has previously established the efficacy of the BWF Shuttle Time program in developing motor competence in children, there are other examples of racquet sport-based studies which have shown promise in enhancing motor skills and other variables in children. For example, Pan et al. [29] reported that a 12 week, twice-weekly intervention based on playing table tennis improved scores on the strength and agility and manual coordination subscales of the BOT-2 motor skills test as well as positively influencing behavioral variables in a sample of boys with attention deficit hyperactivity disorder (ADHD). Likewise, other research by Iserbyt et al. [30] compared the effect of a peer teaching intervention using task cards against a typical teacher focused approach to teaching PE on tennis skill (ball control and stroke technique) in Belgian adolescents. In this seven-week intervention, Iserbyt et al. [30] reported that peer mediated learning facilitated using task cards was comparable to PE lessons taught be a PE teacher for tennis skill performance. Scaling tennis racquets is also reported as advantageous in enhancing the development of tennis skills, with work by Buszard et al. [31] reporting that smaller racquets resulted in significantly better acquisition of forehand and backhand hitting technique, compared to larger racquets, in 6–7-year-olds who undertook a five-week tennis intervention as part of school PE. The teaching games for understanding approach has also been demonstrated as effective in enhancing tennis game-based decision making and skill execution in 9–12-year-olds [32]. While not directly comparable to the intervention employed in the present study, the results of the current study align with the conclusions drawn in the aforementioned research in that racquet sport specific intervention in lieu of school PE is effective in enhancing motor competence and motor skill. Comparing the effectiveness of the different approaches used in prior studies, e.g., teaching games for understanding, peer mediated learning to the method advocated for the BWF Shuttle Time program [15] would be an interesting future study in establishing if it is one specific type of intervention, or simply any kind of racquet sport intervention which is more effective in enhancing children’s FMS.

We deliberately assessed children in key stage 1 and key stage 2 of the English National Curriculum, where the focus for PE in key stage 1 is on the development of FMS, and the focus for key stage 2 is the development of sport-specific skills. Understanding if the BWF Shuttle Time program, which was conceived to be used with children aged 5–15 years, was effective in enhancing FMS, perceived motor competence, and motor fitness is a needed first step in establishing the evidence base for an intervention that is currently employed in over 120 countries worldwide [11]. Where an existing program is used in such a wide age range but without empirical support for its efficacy, a key factor in refining intervention efficacy is determining if the ‘one size fits all’ approach as originally proposed in the Shuttle Time program is effective for children in different stages of development. The current study provides this evidence. Following from the findings presented in the present study further research refining the structure and format of activities undertaken within a Shuttle Time intervention would be welcome in providing greater tailoring of the BWF Shuttle Time program for the needs of children of different ages and at different points on the motor competence pathway.

### Limitations and Direction for Future Research

The current study is not without limitations. Future studies would be welcome which examine if there is a change in physical activity as a consequence of undertaking the BWF Shuttle Time intervention alongside the changes we document related to FMS. Likewise, we could not assess whether there was any effect of being in the control group. It is possible that the control group may have changed their behavior due to the knowledge they were in the control group. By having classes allocated to intervention and control groups we sought to minimize the possibility of such an effect, but future studies should formally control for this possibility. We are also cognizant that the data presented here reflects the effect of a six-week Shuttle Time program undertaken once per week, in lieu of statutory PE. A six-week period was undertaken as prior work [13] has demonstrated this duration of motor competence intervention can be effective and, importantly, fits within the demand of a crowded school curriculum. The 10-week post intervention follow up took place at the end of the next school term for similar reasons although we acknowledge the optimum duration of follow up testing for school-based FMS interventions has yet to be established. The Shuttle Time intervention was also administered following the recommended guidance for teachers as given by the Badminton World Federation [11]. The pedagogic approach of the intervention, although not explicitly stated in the guidance for administration, is primarily based on directed learning with specific cues being provided by teachers to facilitate movement. The intervention was administered by movement trained professionals and intervention fidelity was monitored using time on task. This confirmed that the children were physically engaged in each activity for approximately 45 min of each 50 min intervention session. A process evaluation of the intervention was not possible in the present study. Questions remain in relation to the most effective pedagogic strategy for use in the BWF Shuttle Time and what extent the teacher manual is interpreted by teachers with no prior movement training. Similarly, as the intervention was administered by a movement trained professional but the control conditions were administered by the class teacher, this could be considered a source of bias. Future work evaluating the efficacy of the BWF Shuttle Time program as administered by class teachers would be a key next step in establishing the utility of the BWF program for use in schools.

## 5. Conclusions

To conclude, both process (i.e., the quality of the movement) and product (i.e., the outcome of the movement) FMS scores improved as a result of a six-week BWF Shuttle Time program with changes more prominent in younger (6–7 years old) compared to older (10–11 years old) children. Improvements in FMS post intervention appeared to be retained 10 weeks post intervention. The current study suggests that the BWF Shuttle Time program is beneficial in developing children’s FMS but has a greater effect in children in key stage 1 of the English school curriculum.

## Figures and Tables

**Table 1:** Structure of the 6-week shuttle time program.

Week 1	Week 2	Week 3	Week 4	Week 5	Week 6
Warm-Up (10-min)					
Balance Exercises	Balance Exercises	Balance Exercises	Balance Exercises	Dynamic Balance	Dynamic Balance
Mobility Exercises #1,2, and 4	Mobility Exercises #1,2, and 4	Mobility Exercises #1,2, and 4	Mobility Exercises #1,2, and 4	Mobility Exercises #1,2, and 4	Mobility Exercises #1,2, and 4
Having a Lunge	Having a Lunge	Having a Lunge	Having a Lunge	Having a Lunge	Having a Lunge
	Balance and Throw	Balance and Throw	Balance and Throw	Balance and Throw	Balance and Throw
Main Body (40-min)					
Balloon tap	Balloon tap relay With hand With Racquet	Mirror Chase with Throw and Catch	Mirror Chase with Throw and Catch	Mirror Chase with Throw and Catch	Mirror Chase with Throw and Catch
Balloon tap relay	Mirror Chase with Throw and Catch	Grip Change with Shuttle	Grip Change with Shuttle	Shuttle Chase	Shuttle Chase
Mirror Chase	Grip Change with Shuttle	Balance the Racquet	Throwing Game 1	Forehand and Backhand Lift Merry go Round	Forehand and Backhand Lift Merry go Round
Mirror Chase with Throw and Catch	Balance the Racquet	Throwing Game 1	Backhand Short Serve	Backhand Short Serve	Backhand Short Serve
Balancing Shuttles	Keep your Court Free	Chase and Hit Forehand Backhand	Chase and Hit Forehand Backhand	Flat Play	Flat Play
	Balancing Shuttles	Balancing Shuttles	Balancing Shuttles	Balancing Shuttles	Balancing Shuttles
